# Occupational lead exposure among automotive garage workers – a case study for Jimma town, Ethiopia

**DOI:** 10.1186/1745-6673-7-15

**Published:** 2012-07-09

**Authors:** Yalemsew Adela, Argaw Ambelu, Dejene A Tessema

**Affiliations:** 1 Department of Environmental Health Sciences, College of Public Health, Jimma University, Jimma, Ethiopia; 2 Department of Chemistry, College of Natural Sciences, Jimma University, Jimma, Ethiopia

**Keywords:** Lead, Garage workers, Blood lead level, ‘khat’ (*Catha adulis*), Ethiopia

## Abstract

**Background:**

In Ethiopia, although there are numerous small-scale and medium industries which use lead-based raw materials that may pose health risks to workers, there are no workplace regulations for lead exposure. Moreover, there are no studies carried out on the blood lead levels (BLLs) of workers or on the contribution of common workplace practices to lead poisoning.

**Method:**

A cross-sectional study on the BLLs of 45 automotive garage workers and 40 non-garage workers was carried out in the town of Jimma, Ethiopia. In addition to BLL analysis, data on some risk factors such as smoking, and chewing ‘khat’ (the leaves of *Catha adulis*) were gathered through structured questionnaires and interviews and data analysis was performed using SPSS (version 16). The *t*-test was used to compare mean BLLs of study groups. The analysis of variance (ANOVA), Kruskal-Wallis, Pearson chi-square and odds ratio tests were used to investigate the associations between specific job type, smoking and/or ‘khat’ chewing, service years and occurrence of non-specific symptoms with BLLs.

**Results:**

The mean BLL of the automotive-garage workers was found to be significantly greater than that of the controls. The BLLs of all the lead-exposed individuals were found to be over 10 μg/dL, and 53% of them had BLLs ranging 12 – 20 μg/dL, with the remaining 47% having over 20 μg/dL. The BLL of the workers increased with the duration of working in an automotive garage.

Individuals involved in manual car painting comprise a larger percentage (58%) of those with the highest BLLs (≥ 20 μg/dL). Lead accumulation in individuals who chew ‘khat’ in the work place was found to be faster than in those who are not used to chewing ‘khat’. ‘Khat’ is an evergreen shrub native to tropical East Africa, with dark green opposite leaves which are chewed when fresh for their stimulating effects.

**Conclusion:**

The findings of the study have clearly demonstrated that the BLLs of automotive-garage workers in Jimma town are considerably high with a range of 11.73 – 36.52 μg/dL and the workers are in danger of impending lead toxicity. The BLLs of the workers are influenced by their occupational practices, chewing *Catha adulis* leaves at the workplace, and the time spent working in an automotive garage.

## Background

Lead is one of the most widely distributed toxins in our environment. Although its toxic effects have been known for centuries, occupational exposure to lead that results in poisoning, be it moderately or clinically symptomatic, is still common in many countries of the world [1,2]. Excessive occupational exposure over a brief period of time can cause a syndrome of acute lead poisoning. Clinical findings in this syndrome include abdominal colic, constipation, fatigue and central nervous system dysfunction. With even greater doses, acute encephalopathy with coma and convulsions may occur, whereas in cases of milder exposures, headaches and personality changes may be the only signs of neurologic toxicity [3]. Children are particularly susceptible to lead intoxication that causes various neurological and behavioural problems, ranging from raised hearing threshold to reduction in intelligence quotient (IQ) at low blood lead concentrations. Although no threshold has been determined for the harmful effects of lead in children, a 1991 Centers for Disease Control and Prevention (CDC) Report has put the BLL of concern in children at 10 μg/dL. The level of concern has changed over the past few decades, from 60 μg/dL (1960), to 30 μg/dL (1970), to 25 μg/dL (1985), to 10 μg/dL (1991) [4].

Occupational lead exposure in many developing countries is entirely unregulated, often with no monitoring of exposure [5]. In Ethiopia, although there are numerous small-scale and large industries which use lead-based raw materials that may pose health risks to workers, there are no workplace regulations for lead exposure and no data are available with the labour departments among the workers of small-scale lead-based units with regard to lead poisoning. Many people working for different manufacturing or service rendering organizations such as battery manufacturing workers, gas-station attendants, radiator repair workers, solderers of lead products, and welders, are involved in jobs which expose them to gradual health risks from exposure to lead without having any idea about the materials they are handling. Due to lack of awareness about their exposure, workers usually eat, smoke or drink while at work and such workplace practices may aggravate their exposure [6,7].

In Ethiopia and in some other East African countries, ‘khat’ chewing at the workplace is a common practice. ’Khat’ *(Catha adulis)* is an evergreen shrub native to tropical East Africa, with dark green opposite leaves. The leaves of ‘khat’ are chewed fresh for their stimulating effects. After chewing the leaves, people may swallow the juice and throw away the residue or swallow whatever they chewed. In many work areas, the workers who chew ‘khat’ do so at the workplace. This is typically done by cutting the leaves and putting them into the mouth from time to time while performing duties. Whatever the material is that the workers are handling, they do not wash their hands each time they cut and put the leaves into their mouth. As a result, the various toxic substances, including lead, that have stuck to the hands of these workers might easily get transferred onto the ‘khat’ leaf surface and then ingested with the ‘khat’ by the workers.

Relating the concentration of heavy metals, such as lead, in humans to an environmental and occupational level is crucial in order to determine areas of health risk. Most toxicology studies rely on blood lead level as the measure of exposure [8-10]. Lead in shed deciduous teeth is sometimes quoted being regarded as a record of past lead exposure [11,12]. Other materials that have been used to estimate the amount of lead in human beings include hair [13-15], urine and faeces [16,17].

Auto-garage workers in Ethiopia are involved in car painting, soldering, welding and other repairing activities. The garage compounds in which the workers carry out their daily activities are usually filled with fuel exhaust from automobiles entering or leaving the garage’s compounds. Moreover, workplace ‘khat’ chewing is common practice for many auto-garage workers. Most of the workers have no idea about the toxic metals they might be exposed to; as a result, they pay little attention to protecting themselves from the possible inhalation or ingestion of such toxic substances, nor are they given awareness on the issue or advised to take the necessary protective measures. Despite this fact, no study has been conducted to assess the BLLs of people working in auto-garages or of workers in other industries that are expected to pose health risks to workers. However, a single cross-sectional study on the occupational lead exposure of 51 workers in lead acid battery repair units of transport service enterprises at Addis Ababa, using δ-Aminolevulinic acid (δ-ALA) levels in the urine and serum as a biomarker, has been reported [18].

**Figure 1:**
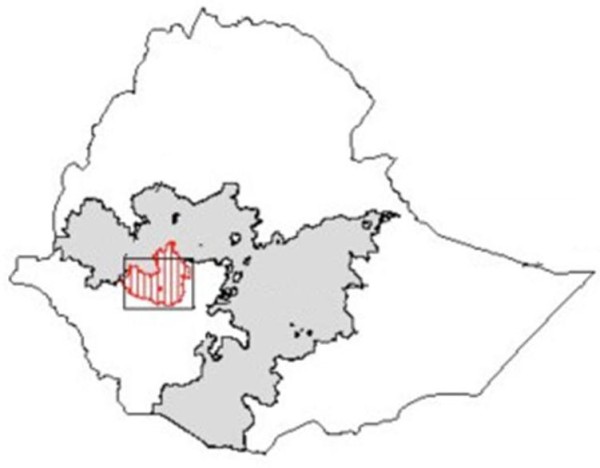
Location of Oromia Regional State (solid shading) and Jimma zone (vertical shading) within Ethiopia.

Jimma is one of the largest towns in Southwestern Ethiopia, located in the Jimma Zone of the Oromia Region (Figure 1). Based on figures from the Central Statistical Agency in 2005, this town has an estimated total population of 159,009 of whom 80,897 were men and 78,112 women [19]. 

According to data obtained from the zone’s Trade and Industry office, there are a total of about 33 small-scale industries involved in furniture production, food processing, metal and woodwork, bakery and pastry, flour**-**making, and coffee processing. There are no large-scale industrial activities in the town which are expected to expose workers to lead pollution. It could also be assumed in Jimma that, despite the continued use of leaded gasoline, a situation where lead emissions from motor vehicles would constitute a serious risk to public health is not anticipated, due principally to the relatively small number of cars in relation to the size of the town. Such a conclusion, however, would not be valid without evidence of completed work.

There are about 23 automotive garages in the town, each of which has an average of 15 workers. All of these garages offer multiple auto-repair services in a single compound. Within this compound, all workers carry out their specific jobs near other colleagues engaged in other activities, moving around to share tools and help one another. Therefore, all the workers are exposed virtually to the same extent to the toxic substances resulting from all the services offered in the auto-garage. The problem of exposure may be further compounded with the chewing of ‘khat’ at the work place. Preliminary observations have revealed that the automotive-garage workers who are used to chewing ‘khat’ while at work are taking the ‘khat’ under poor hygienic conditions and all of them have no idea about the possible toxic substances they might ingest with the ‘khat’ or inhale from the surrounding air. As a result, they use no protective devices to minimize exposure. Therefore, the BLLs of automotive garage workers around Jimma might be higher than other people who are not occupationally exposed. On top of this, auto garage workers who are used to workplace ‘khat’ chewing and smoking might have higher BLLs than their colleagues who are not used to practicing these habits while at work. This study was therefore aimed at investigating the BLLs and associated health problems of automotive garage workers in Jimma and relating the data to workplace practices of chewing ‘khat’ and smoking.

## Methods

### Study subjects and study design

The study was a cross-sectional BLL survey that included blood lead sampling from 45 occupationally exposed garage workers (44 males, 1 female) and 40 controls (36 males, 4 females). The occupationally exposed group included individuals who were mainly involved in manual auto spray-painting or welding for a duration of between 1 to 25 years in the auto-garages where excessive usage of petrol and petroleum by-products takes place with a daily exposure of 8 to 12 hours. The occupationally non-exposed group members were university students and teachers who had apparently no history of lead exposure, were non-smokers, non-khat chewers and non-alcoholics.

### Reagents and laboratory ware

Analytical standard solutions of lead were prepared by serially diluting a 1000 mg/L stock calibration standard solution (Spectro ECON). All chemicals and reagents used were of analytical grade purchased from Merck (Darmstadt) or Sigma Chemical Co.

### Blood sample collection

Venous blood samples (4 mL each) were collected from the 45 garage workers and, 40 apparently healthy non-garage workers using carefully labelled vacutainer tubes containing 7.2 mg K_2_EDTA by qualified medical laboratory professionals. All samples were then preserved at 4°C until digestion.

Blood specimen collection was carried out using separate sterilized needles and gloves for each individual. All used needles and gloves were packed in appropriately labelled disposable bags and taken to the Jimma University Specialized Hospital waste disposal unit.

### Sample preparation

The blood specimens were heated in a hot water bath at 37^°^C for twenty five minutes and homogenized by shaking for one minute. Accurately measured three mL of each of the blood samples was transferred into a Pyrex test tube. A 3:1 mixture of trichloroacetic acid (TCA 5%) and perchloric acid solution (2 M) was added into each test tube and centrifuged for 25 minutes at 3000 r.p.m. The supernatant from each sample was decanted into a labelled sample bottle and the precipitate was further digested with 3.0 mL 2 M perchloric acid and centrifuged for 15 minutes. The supernatant from each centrifuged sample was decanted and mixed with its corresponding supernatant from the first digestion. Finally, the digests were stored at – 4°C until dispatched for analysis.

### BLL analysis

The concentration of lead in the blood samples was determined by Flame Atomic Absorption Spectrometer (NovAA 300) at 283.3 nm after optimizing the various instrument parameters. Triplicate samples were analyzed in each determination and averages of triplicate measurements were taken for each sample. Instrument drift was controlled by running standards after analyzing 10 samples. Quantification of lead in blood was carried out with the help of a standard lead solution. Percentage recoveries determined from blood samples fortified with 10 μg of lead per 4.0 mL of blood sample averaged 94.6%. However, no correction for recoveries was performed in our data.

### Data collection

In addition to determining the concentration of lead in blood samples, data on some risk factors for lead poisoning such as: addiction to alcohol, smoking, ‘khat’ chewing, and eating and/or drinking habits at the workplace, were gathered through questionnaires and interviews. A standardized structured questionnaire, designed to yield information on associated risk factors with the observed BLL, was prepared in English and administered after obtaining consent from the participants of the study. Each item in the questionnaire was interpreted into the local language for those who do not understand English. In addition to the questionnaire, participants were interviewed privately on further points. The interviews included detailed demographic information, exposure history and the presence and nature of lead-related symptoms.

### Statistical analysis

Statistical analyses of results were basically performed by using SPSS (version 16). Comparison of mean BLLs of study groups was carried out using a *t*-test. One-way ANOVA was used to investigate the variation in BLL with the specific job types of the auto-garage workers. The Pearson chi-square statistic and the odds ratio test were used to investigate the associations between BLL and service years, and BLL and occurrence of non-specific symptoms, respectively. The Kruskal-Wallis test was used to investigate the dependence of BLL on smoking and/or ‘khat’ chewing habit in the workplace. All data were expressed as mean ± SD and the level of significance was determined at p < 0.05.

### Ethical consideration

The study was conducted upon obtaining ethical approval by the Jimma University Ethical and Research Committee. The purpose of the study was clearly explained to the study participants following a pre-developed procedure and oral consent was obtained from each of the participating individuals and the auto-garage owners.

Blood specimen collection was carried out using a separate sterilized needle and glove for every individual. All used needles and gloves were packed in appropriately labelled disposable bags and taken to the Jimma University Specialized Hospital waste disposal unit.

## Results and discussion

### BLLs of occupationally exposed and non-exposed groups

The mean lead concentrations of the garage workers and controls are given in Table 1. According to the *t*-test the difference between the mean BLL of the garage workers, 19.76 μg/dL (95% CI: 18.45 – 21.06, median: 19.75 μg/dL; range: 11.73 – 36.52 μg/dL), and that of the controls, 10.73 μg/dL (95% CI: 10.05 – 11.41, median: 10.40 μg/dL; range: 5.6 – 15.64 μg/dL) is significant (p < 0.05).

**Table 1:** BLLs of the garage workers and controls

**Category**	**Mean Pb conc (μg/dL ± SD)**	**95% CI (μg/dL)**	**Range (μg/dL)**	**% BLL ≥ 10 μg/dL**
Garage workers	19.75 ± 4.46	18.45–21.06	11.73–36.52	100
Controls	10.73 ± 2.22	10.05–11..41	5.6–15.64	56

The BLLs of the auto garage workers were found to vary with the specific job type they are involved in. The mean BLL of the workers involved in manual auto painting was 21.12 ± 5.59 μg/dL, that of welders 19.19 ± 4.08 μg/dL, and that of workers involved in both job categories 20.30 ± 4.52. The observed differences, however, were not statistically significant (*p* > 0.05). The BLLs of the garage workers were all greater than 10 μg/dL, while 41% of the controls had BLLs lower than this value. The remaining 59% of the controls had BLLs ranging 10–16 μg/dL. Among the garage workers, 53% had BLLs ranging from 12 to 20 μg/dL, and the remaining 44% of them had 20 to 27 μg/dL. One person among the garage workers had a relatively higher BLL, 36.52 μg/dL, and the person was identified to be an alcoholic, smoker, ‘khat’ chewer, and had served for 25 years in auto garages. The female garage worker who participated in the study had a BLL of 15.87 μg/dL. She had served for over 10 years, and did not chew ‘khat’, smoke or drink alcohol. The mean BLL of the four females among the controls was 10.13 μg/dL (95% CI: 9.36 – 10.90, median: 9.96 μg/dL; range: 9.38 – 11.22 μg/dL).

### BLLs of occupationally exposed group relative to service years

**Figure 2:**
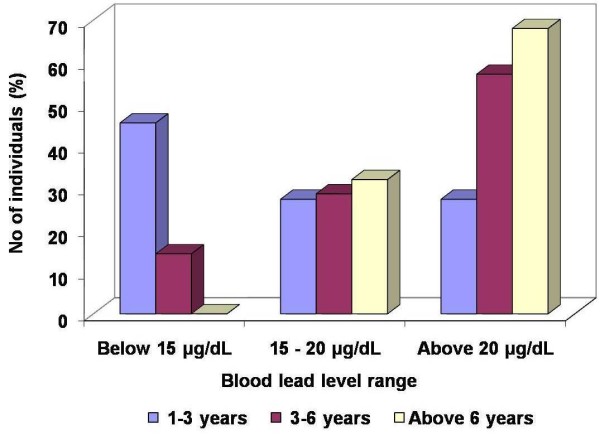
Proportion of the garage workers with BLLs less than 15 μg/dL, between 15 to 20 μg/dL and above 20 μg/dL in the 1 – 3, 3 – 6 and above 6 years of service categories.

The proportion of individuals with BLLs less than 15, 15 to 20 or above 20 μg/dL among the garage workers with service years between 1–3, 3 – 6 and above 6 years are given in Figure 2. The figure clearly shows a steady increase in the proportion of individuals with higher BLLs with an increase in service years. The chi-square test has revealed that the dependence of BLL on service years is statistically significant (*p* < 0.05). Among the individuals in the 1 – 3 service year group, the relative number of individuals with BLLs of less than 15 μg/dL is greater than that of individuals with BLLs ranging from 15 to 20 μg/dL or above 20 μg/dL. Forty-six percent of the garage workers with service between 1 – 3 years and 14% of those with service between 3 – 6 years were found to have BLLs less than 15 μg/dL.

Among the workers with more than 6 years of service, 68% had BLLs above 20 μg/dL, 32% in the range from 15 to 20 μg/dL and none of them had less than 15 μg/dL. Individuals with more than 10 years of service comprise a larger percentage (88%) of those with BLLs above 20 μg/dL. This clearly shows the direct relationship between BLL and service years.

### BLL and smoking/‘khat’-chewing habits

The mean BLL of the total garage workers who were neither smokers nor ‘Khat’ chewers was 16.58 ± 3.5 μg/dL (n = 14) and that of the ‘Khat’- chewing non smokers was 20.17 ± 3.11 μg/dL (n = 25). According to the Kruskal-Wallis test, the observed BLL difference between the two groups is significant (*p* < 0.05). Table 2 illustrates the mean BLLs of the garage workers who were ‘khat’ chewers but not smokers and, non-khat chewers and non-smokers in the service year ranges of 1 to 3, 3 to 6 and above 6 years. As shown in this table, among the 11 individuals with service years ranging 1 to 3 years, the mean BLL of those who were habituated neither to ‘khat’ chewing nor to smoking had a mean BLL of 12.57 ± 0.88 μg/dL (n = 4). However, those who were non-smokers but habituated to ‘khat’ chewing had a mean BLL of 20.19 ± 4.06 μg/dL(n = 7). Six of the seven ‘khat’ chewers had BLLs above 18 μg/dL and only one individual had a BLL of 13.89 μg/dL. The fact that both the ‘khat’ chewers and non-chewers are not smokers and that the BLLs of the non-khat chewers is significantly lower than that of the ‘khat’ chewers indicates that ‘khat’ chewing either accelerates lead accumulation or is an additional source of lead intake. A similar difference between the two groups was not observed in the BLLs of the individuals with more than 3 years of service in the auto garages. The impact of ‘khat’ chewing on lead accumulation steadily decreased with service years, and in individuals with more than 10 years of service its impact was not visible.

**Table 2:** Relationship of BLL with smoking, ‘khat’ chewing, and/or smoking habit and service years

**Service Years**	**‘Khat’ Chewing**	**Smoking**	**n**	**Mean BLL (μg/dL)**	**Range (μg/dL)**	**Median (μg/dL)**	**CI (p = 0.05) (μg/dL)**
1 – 3 years	×	×	4	12.57	11.73–13.8	12.37	11.69–13.45
	√	√	-	-	-	-	
	√	×	7	20.19	13.89–27.1	19.91	16.13–24.25
	×	√	-	-	-	-	
3 – 6 years	×	×	2	18.51	16.51 & 20.51*	-	14.59–22.43
	√	√	1	21.99	-	-	-
	√	×	4	22.04	18.21–25.94	22	18.87–23.21
	×	√	-	-	-	-	-
Above 6 years	×	×	8	18.94	15.87–21.68	19.61	17.25–20.63
	√	√	3	25.46	19.58–36.52	20.29	14.63–36.3
	√	×	14	19.63	15.66–23.69	19.06	18.29–20.97
	×	√	2	25.16	24.08 & 26.23*	-	23.06–27.26

n = number of workers, Х = Not smoking or ‘Khat’ chewing, √ = Smoking or 'Khat' chewing.

*where n = 2, both blood lead concentrations are given in place of the range.

### Lead toxicity symptoms

The odds ratio of the reported non-specific symptoms in the garage workers in relation to the controls was calculated and the results obtained are shown in Table 3. The results clearly show that among the reported non-specific symptoms, the occurrence of wrist drop, tingling and numbness in fingers and hands, nausea, and decreased libido in the auto garage workers are significantly greater than in the controls.

**Table 3:** Reported symptoms among the occupationally exposed (n = 45) and the controls (n = 40) and the ratio of their odds

**№ of ‘Yes’ response for symptom**				
**Symptom**	**Cases**	**Controls**	**Odds Ratio**	**p-value**
Depression	28	7	7.76*	0.00
Memory impairment	13	6	2.30	0.21
Sleep disturbance	23	9	3.60*	0.01
Concentration difficulty	9	11	0.66	0.32
Headaches	17	14	1.13	0.91
Wrist drop	25	1	48.75*	0.00
Tingling & numbness in fingers/hands	12	1	14.18*	0.01
Lack of appetite	12	5	2.55	0.18
Nausea	10	1	11.14*	0.02
Constipation	10	3	3.52	0.13
Abdominal discomfort	16	8	2.21	0.17
Decreased libido	21	3	10.79*	0.00

* significant relative risk of occurrence in the auto garage workers at p < 0.05.

**Figure 3:**
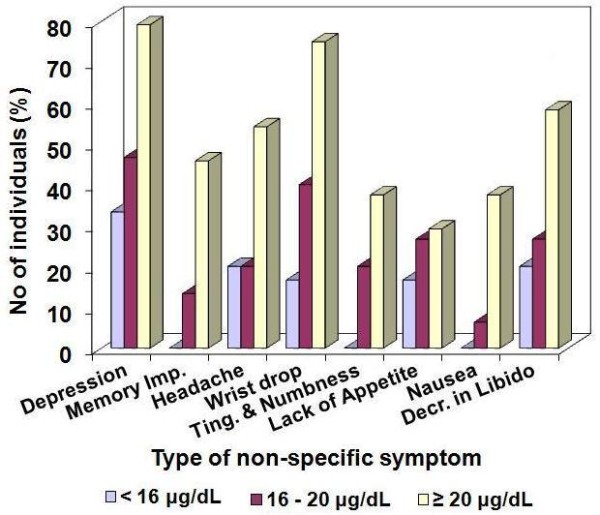
Non-specific symptoms observed at different BLL.

The proportion of individuals affected by the non-specific symptoms among the individuals with BLLs: less than 16, 16 to 20 or above 20 μg/dL, was assessed and the results obtained are illustrated in Figure 3. The results clearly indicated an increase in the prevalence of all the symptoms with an increase in BLL.

Among the symptoms assessed, depression, wrist drop, and decreased libido were the most prevalent ones in the individuals with BLLs ≥ 20 μg/dL. About 80% of the garage workers in this BLL range reported having symptoms of depression, 75% for wrist drop and 58% for decreased libido.

Results of the odds ratio test for the relative occurrence of the non-specific symptoms between the garage workers with BLLs of less than 20 μg/dL (n = 25) and the controls (n = 40) are given in Table 4. The results clearly show that the occurrence of most of the symptoms in the garage workers is significantly greater than in the controls (*p <* 0.05). This could be a clear indication for the negative health impacts of BLLs as low as 10 μg/dL.

**Table 4:** Reported symptoms among the auto garage workers with BLLs less than 20 μg/dL (n = 25) and the controls (n = 40) and the ratio of their odds

**Yes responses for symptoms**				
**Symptoms**	**Cases**	**Controls**	**Odds ratio**	**p**
Depression	28	7	5.11*	0.02
Memory impairment	13	6	1.08	0.61
Sleep disturbance	23	9	3.18*	0.01
Concentration difficulty	9	11	0.50	0.55
Headaches	17	14	0.66	0.52
Wrist drop	25	1	26.00*	0.00
Tingling & numbness in fingers/hands	12	1	7.43*	0.04
Lack of appetite	12	5	2.20	0.06
Nausea	10	1	5.32*	0.04
Constipation	10	3	2.25	0.16
Abdominal discomfort	16	8	1.88	0.29
Decreased libido	21	3	8.22*	0.00

* significant relative risk of occurrence in the auto garage workers at p < 0.05.

During interviews, the garage workers reported some non-specific symptoms which were not included in the questionnaire. Among the workers, 15 (33.3%) reported having developed a feeling of metallic taste in their mouth, 9 (20%) reported having blurred vision, and 11 (24.4%) had dry white scars in one or two areas on their heads.

## Discussion

Occupationally related BLL assessment has not previously been carried out in any part of Ethiopia. However, in a cross-sectional study carried out in Addis Ababa on lead exposure among storage battery repair workers by measuring urinary aminolevulinic acid levels, higher levels of urinary aminolevulinic acid were found in the storage battery repair workers and the possible parallel rise in BLLs of the workers was predicted. The results obtained in our study have shown that auto-garage workers have significantly greater BLL than the non garage workers (*p* < 0.05). This clearly indicates that auto-garage workers are more likely to be exposed to lead due to occupational incidences than the general population. Furthermore, the results obtained in our study are consistent with the results of other studies carried out on the determination of the BLLs of: ninety-seven occupationally and non-occupationally exposed individuals in Nigeria [20], workers involved in various types of jobs in the United Arab Emirates [21], thirty one male non-smoking industrial workers in Iran [22], and apprentices working in lead-related industries in Turkey [6]. Among the lead-exposed garage workers, the mean BLL of individuals who were mainly involved in manual auto painting (21.12 ± 5.59 μg/dL) was slightly higher than that of the mechanics (19.19 ± 4.08 μg/dL). Comparison of the mean values by using a *t*-test has shown that the observed difference was not, however, statistically significant. A study done in Bangkok on 52 mechanics, 27 dye sprayer and 20 controls, reported mean BLLs of 8.7 μg/dL, 12.02 μg/dL and 6.63 μg/dL, respectively [23]. The mean BLLs obtained by these researchers for all the three groups were much lower than those obtained in our study. The relative difference between the BLLs of the mechanics and the auto-painters in their study (27.6%), however, is greater than that of the difference obtained in our study (9.1%). The observed higher BLL in the painters than in the mechanics might indicate a greater exposure of the dye sprayers relative to the mechanics. The painters, in addition to the oral exposure routes, are more likely exposed to inhalation of lead fumes found in the dyes than those workers engaged in other auto-repairing activities. This could be a possible reason for the observed BLL difference between the two groups.

The garage workers were found to exhibit significantly higher levels of the non-specific symptoms which included: depression, sleep disturbance, wrist drop, tingling and numbness in fingers and hands, nausea, and decrease in libido relative to the controls. Moreover, the prevalence of these symptoms was higher in the workers with higher service years than in those with lower service years. Comparison of the prevalence of the non-specific symptoms between the occupationally exposed individuals with BLLs less than 20 μg/dL (n = 25) with that of the controls (n = 40) has also revealed that there is a significantly greater prevalence of most of the symptoms in the garage workers. The Association of Occupational and Environmental Clinics (AOEC) has revealed the health effects of various BLLs on lead-exposed adults, and according to this document, the non-specific symptoms such as: headache, sleep disturbance, fatigue, and decreased libido are shown to occur in the BLL range between 20 and 39 μg/dL [24]. However, the findings of our study suggest that these symptoms are exhibited by lead-exposed individuals at lower BLLs (10 – 20 μg/dL) than indicated in the AOEC document. Our report on the variations of the non-specific symptoms between the two groups is entirely from what the two groups revealed in the questionnaires and interviews. Although this may be suggestive of the adverse effects of lead (Pb) on the exposed individuals relative to the non-exposed, a close medical investigation is required to affirm that the epidemiologic variations between the two groups are exclusively results of the difference in the BLLs of the groups.

‘Khat’ chewing has been found to enhance lead accumulation in the first 1 – 3 years of service in the occupationally exposed individuals. The mean BLL of the ‘khat’ chewers in the 1 – 3 service year range was 61% higher than the mean BLL of the non-chewers in the same service year range. The observed elevated level of lead in the ‘khat’ chewers could most likely be due to oral ingestion. The garage workers are chewing ‘khat’ at the workplace. Moreover, they chew the ‘khat’ while carrying out their work and do not wash their hands each time they cut the leaves and put them in their mouth. This makes lead entry into the digestive system easier, thereby increasing BLL.

Several potential limitations of our study may have affected the analysis. The records of environmental Pb exposure in the proximity of the auto-garages were not available because monitoring of Pb in air was not enforced. Any observed difference in response to occupational and environmental Pb exposure may, therefore, be attributed to a degree of exposure to Pb. The participants in the control group were selected from university students and teachers. As a result, absence of some epidemiological symptoms in this group might not be exclusively attributed to lower BLL relative to the automotive-garage workers.

## Conclusion

The BLLs of automotive-garage workers in Jimma town are noticeably high with a range of 11.73 – 36.52 μg/dL and the workers are in danger of impending lead toxicity. The BLLs of the workers are influenced by their occupational practices and roughly paralleled with the duration of occupational lead exposure. Workplace ‘khat’ chewing and lack of awareness about the ill health effects of lead and the routes through which it enters the human body has contributed to the easy entry of lead into the body of the workers and the resulting elevated BLL. Further large-scale screening and regular monitoring of auto-garage workers is urgently needed to reduce long term adverse effects of lead exposure.

## Competing interests

The authors declare that they have no competing interests.

## Authors’ contributions

YA carried out the sampling, analytical work and statistical analysis, AA participated in the design and coordination of the study and the statistical analysis. DAT conceived the study, participated in its design and coordination and prepared the manuscript. All authors read and approved the final manuscript.
